# Prediction of antibiotic resistance in *Escherichia coli* from large-scale pan-genome data

**DOI:** 10.1371/journal.pcbi.1006258

**Published:** 2018-12-14

**Authors:** Danesh Moradigaravand, Martin Palm, Anne Farewell, Ville Mustonen, Jonas Warringer, Leopold Parts

**Affiliations:** 1 Wellcome Sanger Institute, Wellcome Genome Campus, Hinxton, Cambridgeshire, United Kingdom; 2 Center for Computational Biology, Institute of Cancer and Genomic Sciences, University of Birmingham, Birmingham, United Kingdom; 3 Department for Chemistry and Molecular Biology, University of Gothenburg, Gothenburg, Sweden; 4 Centre for Antibiotic Resistance Research at the University of Gothenburg, Gothenburg, Sweden; 5 Organismal and Evolutionary Biology Research Programme, Department of Computer Science, Institute of Biotechnology, University of Helsinki, Helsinki, Finland; 6 Helsinki Institute for Information Technology HIIT, Helsinki, Finland; 7 Department of Computer Science, University of Tartu, Tartu, Estonia; University of Technology Sydney, AUSTRALIA

## Abstract

The emergence of microbial antibiotic resistance is a global health threat. In clinical settings, the key to controlling spread of resistant strains is accurate and rapid detection. As traditional culture-based methods are time consuming, genetic approaches have recently been developed for this task. The detection of antibiotic resistance is typically made by measuring a few known determinants previously identified from genome sequencing, and thus requires the prior knowledge of its biological mechanisms. To overcome this limitation, we employed machine learning models to predict resistance to 11 compounds across four classes of antibiotics from existing and novel whole genome sequences of 1936 *E*. *coli* strains. We considered a range of methods, and examined population structure, isolation year, gene content, and polymorphism information as predictors. Gradient boosted decision trees consistently outperformed alternative models with an average accuracy of 0.91 on held-out data (range 0.81–0.97). While the best models most frequently employed gene content, an average accuracy score of 0.79 could be obtained using population structure information alone. Single nucleotide variation data were less useful, and significantly improved prediction only for two antibiotics, including ciprofloxacin. These results demonstrate that antibiotic resistance in *E*. *coli* can be accurately predicted from whole genome sequences without *a priori* knowledge of mechanisms, and that both genomic and epidemiological data can be informative. This paves way to integrating machine learning approaches into diagnostic tools in the clinic.

## Author summary

One of the major health threats of 21st century is emergence of antibiotic resistance. To manage its human health and economic impact, efforts are made to develop novel diagnostic tools that rapidly detect resistant strains in clinical settings. In our study, we employed a range of powerful machine learning tools to predict antibiotic resistance from whole genome sequencing data for *E*. *coli*. We used the presence or absence of genes, population structure and isolation year of isolates as predictors, and could attain average precision of 0.92 and recall of 0.83, without prior knowledge about the causal mechanisms. These results demonstrate the potential application of machine learning methods as a diagnostic tool in healthcare settings.

## Introduction

Antibiotic resistance has turned into an acute global threat. The rise of bacterial strains resistant to multiple antibiotics is expected to dramatically limit treatment effectiveness [1], leading to potentially incurable outbreaks. In addition to new drug development efforts, there is an urgent need for preclinical tools that are capable of effective and rapid detection of resistance [2, 3], as culture-based laboratory diagnostics test are usually time consuming and costly [3].

To accelerate the diagnosis, genetic tests have been devised to identify known resistance genes. The increasingly affordable and available whole genome sequencing data from clinical strains has helped to robustly identify antibiotic resistance determinants, and to curate them in dedicated databases [4, 5]. Given sequence from a new strain, computational methods can then look up known causal genes in these resources [5, 6]. Whilst such rule-based models are highly accurate for some common pathogens with well-characterized resistance mechanisms (e.g. *Mycobacterium tuberculosis* and *Staphylococcus aureus*) [7], they cannot be employed to detect resistance caused by unknown mechanisms in other major pathogenic strains, and require regular curation to remain effective.

Prediction approaches based on machine learning have the potential to overcome these restrictions of rule-based tests. As general-purpose methods, they are agnostic to the causal mechanisms, and learn useful features directly from data [8–11]. Already, decision tree based models have proven valuable for predicting resistance and pathogen invasiveness from genomic sequences [12–16]. However, these studies were limited in both the genetic features used and the methods applied. In particular, both population structure and accessory genome content could contain predictive information, as resistance determinants may be transferred horizontally from other strains, or inherited vertically from an ancestor [2]. Further, the powerful deep learning methods that can utilize complex features interactions were not examined.

Here, we systematically evaluate the performance of machine learning algorithms for predicting antibiotic resistance from *E*. *coli* whole genome sequence data. We present genome sequences and resistance measurements of 255 new isolates and consider them together with published data from recent large-scale studies, as well as simulated datasets. We test whether prediction accuracy improves with including temporal data, population structure, and accessory genome content, and assess how a range of population parameters, such as mutation and recombination rates, influence predictions.

## Methods

### Isolates

We used 1681 strains from four large-scale clinical and environmental *E*. *coli* collections, with available data on the year of isolation, drug susceptibility phenotypes, and whole genome sequence [17, 18]. Furthermore, we collected 255 strains from a range of ecological niches: hospital sewage and water treatment plant from Sweden (Carl-Fredrik Flach); human clinical isolates isolated in Pakistan, Syria, Sweden and USA (Culture Collection University of Gothenburg); a collection of strains producing extended-spectrum β-lactamases isolated in Sweden (Christina Åhrén) and environmental samples from Belgium (Jan Michiels).

### Antimicrobial susceptibility testing

Antimicrobials tested for the newly sequenced genomes included beta-lactams (penicillin: ampicillin (AMP, C.B. (clinical breakpoint): 6μg/ml); cephalosporins: cefuroxime (CXM, C.B.: 8μg/ml), cefotaxime (CTX, C.B.: 4μg/ml), cephalothin (CET, C.B.: 20μg/ml) and ceftazidime (CTZ, C.B.: 0.25μg/ml)), aminoglycosides (gentamicin (GEN, C.B.: 4μg/ml) and tobramycin (TBM, C.B.: 8μg/ml)), and fluoroquinolones (ciprofloxacin (CIP, C.B.: 1μg/ml)). Besides these antibiotics, antimicrobial susceptibility testing results were available for amoxicillin-clavulanate (AMC), amoxicillin (AMX) and trimethoprim (TMP) for the previously sequenced genomes and were used in this study. Antibiotic abbreviations were adopted from the British Society of Antimicrobial Chemotherapy (www.bsacsurv.org/science/antimicrobials). Concentrations used were determined by performing a 2-fold serial dilution, starting from twice the concentrations listed by the European Committee on Antimicrobial Susceptibility Testing (EUCAST) on 25/01/2017, until no growth was observed after 16 hours for the common lab strain BW25113 [19] used as a control in the experiments. In defining resistance, we designated intermediate strains as resistant.

### Sequencing data generation

We extracted DNA with the Bacterial Genomic DNA Isolation 96-Well Kit (Norgen Biotek) as detailed in the manufacturer’s instructions. Libraries were prepared with standard Illumina DNA sequencing library preparation protocols, and sequenced on Illumina HiSeq X with 150 bp paired end reads, multiplexing 384 samples per lane, and achieving average depth of coverage of 40-fold. We used Kraken, which accurately assigns taxonomic labels to the short DNA reads [20], to confirm the presence of *E*. *coli* reads in the pool. The raw sequences for the sequenced data in this study have been deposited in the European Nucleotide Archive (ENA) under the accession numbers described in S1 Table.

### Pan-genome determination

Paired-end reads for the isolates sequenced both here and previously were assembled with Velvet [21] and put through an improvement pipeline [22]. In order to reconstruct the pan-genome, we used the output assemblies and annotated these with Prokka [23]. The annotated assemblies produced by Prokka were then used as input for Roary [24] to build the pan-genomes with the identity cut-off of 95%. In the process of pan-genome construction, coding regions were extracted from the annotated assemblies and then converted to protein sequences (partial coding sequences were filtered out). Roary yielded clusters of homologous gene groups and produced a matrix for the presence and absence of ortholog accessory genes. The variant sites (SNPs) in the core genome alignment were extracted with a SNP sites tool (www.github.com/sanger-pathogens/snp-sites). To visualize the phylogenetic tree with the associated metadata, we used iTOL [25]. The pan-genome data, including sequences of the gene ortholog families, as well as the annotated assemblies for strains are provided in Github (www.github.com/DaneshMoradigaravand/PanPred).

### Population structure calculation

We mapped the short reads to the reference EC958 genome sequence [26] as detailed in [27], and calculated the pairwise SNP distance (number of differing sites) for the core genome alignment of strains with functions in the ape package in R [28]. We identified clusters within the population using a distance-based method in the adegenet package [29]. We clustered sequences using the sequence distance metric with the adegenet package for all possible number of clusters from 1 to number of strains. To this end, we employed the gengraph function in the package that produces graphs from genetic distances, in which pairs of strains are connected if their genetic distance is less than a given cut-off. We considered each connected component of the obtained graph as a cluster. Based on these clusterings, we constructed the population structure matrix S, where s_ij_ = *k* if strain *i* belongs to cluster *k* in the clustering with at most *j* clusters.

### Simulated datasets

To evaluate the performance of prediction tools, we simulated pan-genomes with the simulation script in the Scoary package [30] (https://github.com/AdmiralenOla/Simulate_pan_genome). The simulation process begins with a single genome with 3000 core and 6000 accessory genes that undergoes duplication and gene loss/gain in every generation, and continues until a desired number of genomes is reached; we tested population sizes of 130, 260, 650 and 1300. We examined penetrances, defined as the probability of acquisition/loss of the resistance phenotype simultaneously to the acquisition/loss of the causal resistance gene, of 0.5, 0.6, 0.7, 0.8, 0.9 and 1.

### Feature calculation

We examined different predictors as inputs: 1) matrix of the presence-absence of accessory genomes within the pan-genome (G), where g_ij_ is 1 if gene *i* is present in strain *j*, and 0 otherwise; 2) matrix of population structure inferred from core-genome (S) defined above, and one-hot encoded 3) matrix of SNP sites (SNP), where SNP_ij_ = 0 if strain *j* carries the consensus allele at site *i*, and 1, 2, 3, 4, 5 if it contained A, T, C, G nucleotide or missing information, respectively; 5) matrix of indels (indel), where indel_ij_ = 0 if strain *j* has the reference sequence at site *i* and 1 if it contained any indel and 6) matrix of years of isolation (Y). We standardized each feature to have 0 mean and unit variance. Genes, strain clusters, and SNPs with identical indicator pattern were collapsed, so there are no duplicate rows in the G, S, SNP matrices.

### Resistance prediction

We performed prediction using various combinations of input matrices using resistance indicator as the output. We employed 80% of the data for training and tuning the various models, using 4-fold cross-validation to select the best parameters for each model class according to mean accuracy. The performance of the selected model was then assessed on the remaining 20% held-out data. Positive and negative corresponded to the resistant label.

### Four different models were used along with a rule-based baseline

Logistic regression with L_2_ regularization. We employed the “LogisticRegression” function in the Scikit-learn python package (www.scikit-learn.org) [31], with the “lbfgs” solver, and varied the regularization parameter strength from 0 to 1 with step size 0.01.

#### Random forest classifier

We employed the “RandomForestClassifier” function in Scikit-learn. We varied key parameters including the number of trees in the forest (n_estimators; 100, 600, 2000, 5000) and number of features considered for splitting at each leaf node (max_features), i.e. when searching for the best split, we used total number, as well as square root and binary logarithm selection of the number of features. We also changed parameters controlling the size of tree, including values for minimum number of samples required at a leaf node (min_samples_leaf) (values: 1,5), minimum number of samples required to split an internal node (min_samples_split) (values: 2, 3). We also changed the maximum depth (max_depth) of the tree by assuming that nodes are expanded until all leaves are pure or until all leaves contain less than 10 and 50 strains. We used bootstrap samples for building trees, out-of-bag samples to estimate accuracy, and Gini impurity as the criterion for the information gain.

#### Gradient boosted decision trees

We used the “GradientBoostingClassifier” implementation in Scikit-learn, with learning rate 0.1, and 300, 600 and 5000 boosting stages, and deviance loss. We aimed to make the parameters considered for random forests and gradient boosted decision trees as similar as possible. Therefore, we used the hyperparameter values for the decision tree parameters shared with random forests, including max_features, min_sample_leaf and mean_samples_split. In order to assess the robustness of feature importance analysis, we repeated the optimization with 50 random seeds. To account for overfitting, we also ran iterations with a fraction of 0.8 of samples for fitting the individual base learner models. This turned the model into a stochastic gradient boosting model. As a measure for feature importance, we counted the number of times a feature used in optimization, as well as the average feature rank and importance across multiple replicates.

#### Deep neural networks

We employed the keras library in python (www.keras.io) to build fully connected deep neural networks. We tested various network topologies, including two, four and six layer networks, with two output nodes corresponding to resistant and susceptible states, and 100 and 150 nodes in each internal layer and 200, 400 and 600 nodes for the first layer. We used Adam to train for 20 epochs, with batch size of 128, learning rate of 0.1, drop-out of 0 and 0.2, and stopping when the validation set performance decreased. Due to the small training dataset size compared to the number of features, for ~50% of runs the loss in the validation did not decrease by the end of training the network. We randomly partitioned the data into training (56%), validation (14%) and test (30%) sets, and trained models with different parameters on the training set, evaluated quality on the validation set, and final performance on the test set.

#### Rule-based baseline

We compared our results with a rule-based method based on the detection of known resistance genes. To this end, we employed srst2 [32] and mapped short reads to the ResFinder and the Comprehensive Antibiotic Resistance Database (CARD) of known resistance genes in the srst2 package, using the cut-off of 60% for the length coverage. We checked the ESBL and inhibitor-resistance status (for AMC resistance) of beta-lactamases according to the Lahey hospital and medical center database (www.lahey.org/Studies) and CARD. For aminoglycosides, variants of genes encoding aminoglycosides modifying enzymes were considered as resistance. For trimethoprim, variants of dihydrofolate reductase genes were assumed to cause resistance. Finally, for ciprofloxacin, the *qep* and *oqx* genes, belonging to major facilitator superfamily transporters, were considered as resistance genes.

Details of the performance of each hyperparameter set, as well as distribution of known resistance genes (from CARD and Resfinder), are provided in the Github directory (www.github.com/DaneshMoradigaravand/PanPred). Prediction results for the best performing model are detailed in S3 Table.

## Results

Our data comprise 1936 samples that have been full genome sequenced, and phenotyped for resistance of 11 antibiotics. Resistance was distributed both within specific clades as well as emerging sporadically on divergent lineages (S1 Fig), with an average frequency of 0.35 per drug (range: 0.15–0.63). This pattern suggests both vertical and horizontal spread of resistance determinants. Genome sequences were processed to give gene and polymorphism presence information (1,390 core genes present in >99% of lineages, 90,261 genes present in more than one lineage, 1,432,145 variable sites in core genes), and 1,071 population structure features.

**Fig 1:**
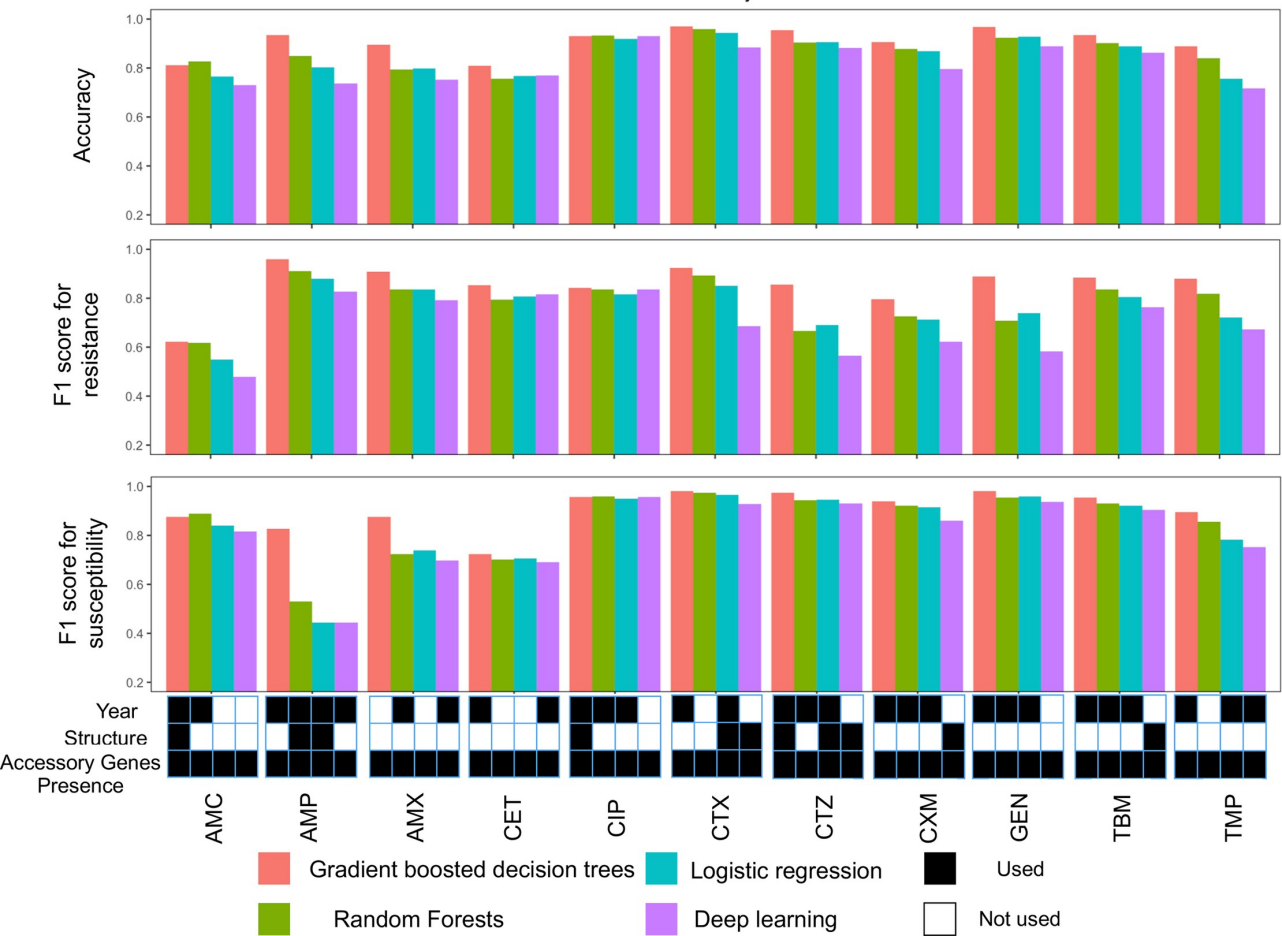
Prediction performance of the best tuned models. Accuracy and F1 score (harmonic mean of precision and recall; y-axis) for resistant (top panel) and susceptible (middle panel) phenotypes for four predictive models (red: gradient boosted decision trees; green: logistic regression; teal: random forests; purple: deep learning) across eleven antibiotics (x-axis). The best model of each class for every drug (x-axis) was identified based on the accuracy for predicting resistance and employed a number of possible combinations of gene presence, population structure, and year of isolation (lower panel; black: feature used; white: feature not used).

We used these input features to test the ability of four machine learning models—logistic regression, random forests, gradient boosted decision trees, and deep neural networks—to predict antibiotic resistance. We varied model hyperparameters, as well as input data types, to establish the most accurate models within each class (Methods). Results were similar on the training and test datasets, demonstrating lack of bias in their choice (S2 Fig). Gradient boosted decision trees performed best for predicting resistance of 11/11, and susceptibility of 9/11 drugs (Fig 1), with average precision of 0.93 and recall of 0.83 (S3 Fig, Table 1). Perhaps surprisingly, deep learning models that account for complex non-linear relations amongst features did not provide substantial improvement over the simpler logistic regression models, or random forests (Fig 1).

**Table 1:** Prediction metrics on held out data for the best performing gradient boosted decision trees model.

Antibiotic	TN	FP	FN	TP	S.PRC	S.RCL	R.PRC	R.RCL	S.FSc	R.FSc	ACC
**AMP**	**24**	**5**	**5**	**118**	**0.83**	**0.96**	**0.83**	**0.96**	**0.83**	**0.96**	**0.93**
**AMX**	**80**	**8**	**15**	**115**	**0.84**	**0.93**	**0.91**	**0.89**	**0.87**	**0.91**	**0.89**
**AMC**	**221**	**34**	**29**	**52**	**0.89**	**0.60**	**0.87**	**0.64**	**0.87**	**0.62**	**0.81**
**CTZ**	**309**	**4**	**13**	**50**	**0.96**	**0.92**	**0.99**	**0.80**	**0.97**	**0.85**	**0.95**
**CTX**	**281**	**5**	**6**	**66**	**0.98**	**0.93**	**0.98**	**0.922**	**0.98**	**0.92**	**0.97**
**CXM**	**273**	**11**	**24**	**68**	**0.92**	**0.86**	**0.96**	**0.74**	**0.94**	**0.79**	**0.91**
**CET**	**38**	**12**	**17**	**85**	**0.70**	**0.88**	**0.76**	**0.83**	**0.72**	**0.85**	**0.81**
**GEN**	**316**	**1**	**11**	**48**	**0.97**	**0.98**	**0.99**	**0.81**	**0.98**	**0.89**	**0.97**
**TBM**	**104**	**3**	**7**	**38**	**0.94**	**0.92**	**0.98**	**0.84**	**0.95**	**0.89**	**0.93**
**TMP**	**73**	**7**	**10**	**62**	**0.88**	**0.90**	**0.91**	**0.86**	**0.90**	**0.88**	**0.89**
**CIP**	**281**	**10**	**16**	**69**	**0.95**	**0.87**	**0.97**	**0.81**	**0.95**	**0.84**	**0.93**

TN: true negatives, FN: false negatives, FP: false positives, TP: true positives, PRC: precision, RCL: recall, S: susceptibility, R: resistance, ACC: accuracy for resistance.

Knowledge of what features that aid prediction will help prioritize data collection and diagnostic efforts. The gene presence and absence predictor (G) was used in all the best predictive models for each of the considered methods (Fig 1; lower panel). This is not surprising, given multiple known resistance mechanisms driven by accessory gene content, e.g. for beta-lactams and aminoglycosides. Population structure information (S) and year of isolation data (Y) were also used in 11 and 30 out of 44 best models, respectively. Adding gene presence to population structure features, with or without the year of isolation, improved the accuracy score by 0.12 on average (S4 Fig). In contrast, once gene presence had been accounted for, there was limited performance gain when including population structure features (S3 Fig). This suggests that accessory gene content already contains information about population structure, which reflects the pattern of polymorphisms in the core genome. Indeed, core genome distance and accessory gene difference matrices are not independent (p < 0.01, Mantel test), which is likely explained by accessory genes acquired by clade ancestors, followed by limited turnover.

Next, we asked which individual features are most frequently utilized. We measured feature importance as the number of times it was used for gradient boosted decision trees, the best performing method, across 50 random fitting replicates on fixed training date (S5 Fig). In general, known resistance genes were identified as the most important, and were most frequently used features for predicting resistance to beta-lactams and aminoglycosides, e.g. ESBLS, including variants of *blaTEM-1* and *blaCTX-M-15*, and extra copies of *ampC* and *phnP* efflux pump genes (S2 Table). For example, the known beta-lactamase *bla-CTX-M* gene ranked first in all models for predicting resistance to beta-lactam ceftazidime, which followed by some genes with unknown function and *ampC* (S5A Fig). Further to known genes, genes that are tightly linked to causal resistance genes may be identified as important. The *group_17190* gene, highly ranked for cefuroxime and cefotaxime was linked to *blaCTX-M-15* (S2 Table).

For 10 out 11 drugs the year of isolation was used in the best performing model. However this feature did not improve accuracy when added to the structure and genetic features for nearly all drugs expect ampicillin and to lesser extend cephalothin (S4A Fig). This was indicated by the temporal distribution of the data, where all the strains collected in 2015 were resistant to these antibiotics. These findings demonstrate that although known resistance genes were most predictive, other features, i.e. year of isolation, may be reproducibly utilized for prediction as well (S5B Fig). Nevertheless, it is clear that the inclusion of some features, such as collection year, reflects bias in the training data rather than biological importance. Time of isolation is informative when an outbreak of multi-drug resistant strain occurs at a limited time period. While not mechanistically informed, time of isolation, and perhaps other clinical and epidemiological features may be integrated and utilized if they robustly improve performance.

**Fig 2:**
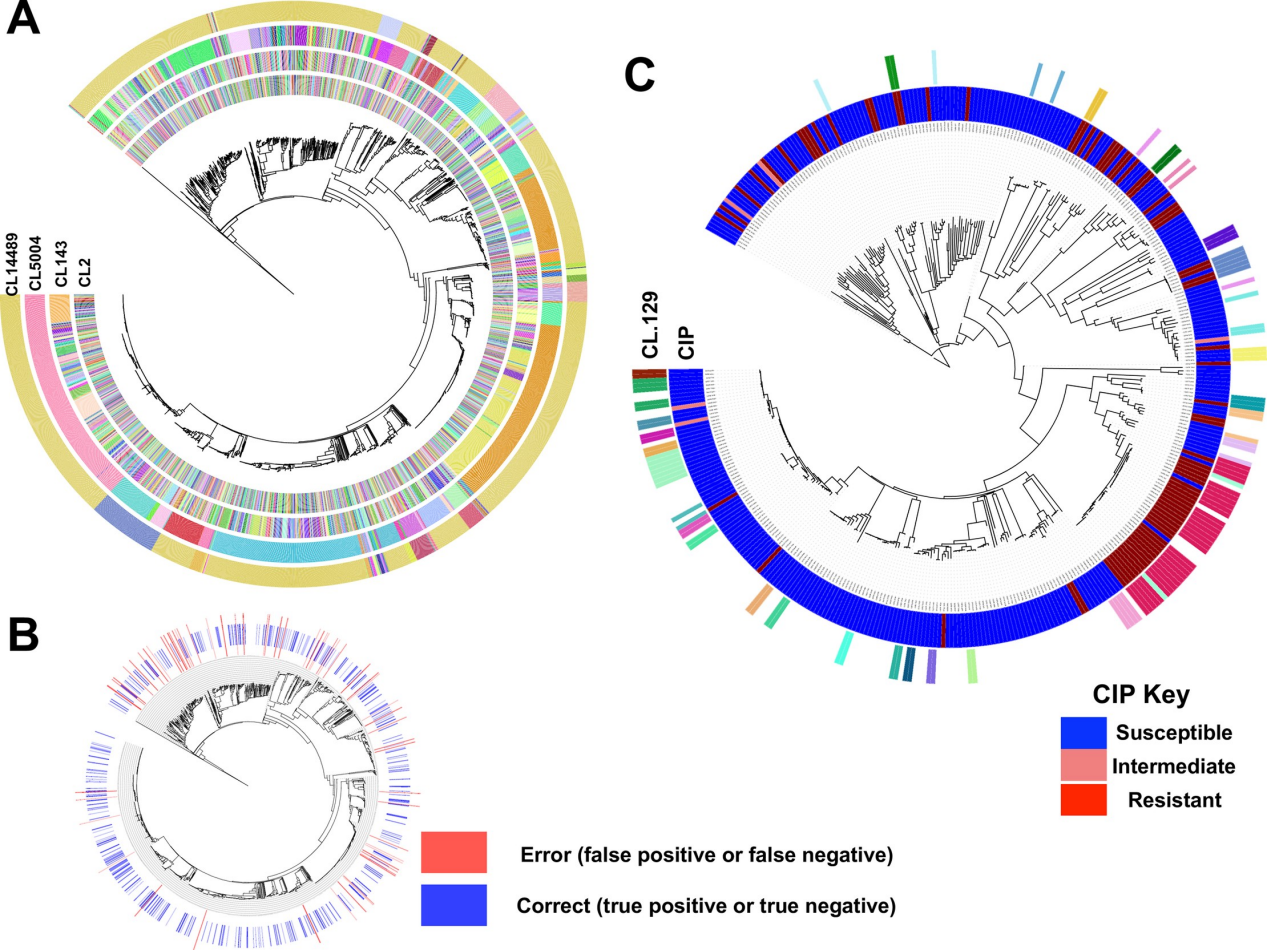
Population structure and phenotypic distribution of the input data. A) Phylogenetic distribution of clusters identified in the population for SNP distance cut-off values of 2, 143, 5054 and 14489 (outer circles) relative to the phylogenetic tree. B) Phylogenetic distribution of correct calls (true positives, true negatives) and errors (false positives, false negatives) when predicting cephalothin (CIP) resistance with the best performing gradient boosted model. The accuracy for resistance was 0.91. C) Phylogenetic distribution of the most important identified population structure feature, clustering with SNP cut-off of 129 (outer ring), compared with the phylogenetic distribution of resistance phenotype (inner ring; blue: susceptible; light red: intermediate and red: resistant) on the test dataset. Clusters with more than one member are shown.

Population structure information was less often selected than gene presence and absence and year of isolation in the best performing models (Fig 1). Nevertheless, training only on population structure produced an average accuracy of 0.79 (range: 0.65–0.91), and this performance could not be achieved with randomized phenotypes (S6 Fig). Population structure features capture both recently diverged and deep clades (Fig 2A), and features included in the models were not limited to a single lineage or common depth (e.g. Fig 2B). As an example, CL129 distinguishes clusters by positing a maximum pairwise sequence distance of 129 nucleotides between isolates, and was identified as the most important feature. Cluster membership at this level of similarity informs of resistance, as 73% of clusters with at least two strains contained either only resistant or only susceptible strains (Fig 2C). In these cases, resistance status of an ancestral strain of the clades was likely retained in descendants and did not change due to horizontal gene transfer, mutation, or sporadic gene loss. Altogether, the results show that predictive models can utilize genetic relatedness and population structure for predicting resistance, as has been observed in traditional eukaryotic genetics [9].

Due to the correlation between the pan-genome and structure data, determining the relative contribution of gene content to prediction is not straightforward (see Discussion). In order to obtain insights into this, we assessed the performance of the model on a new lineage. To this end, we used the ST131 lineage as test dataset, and the rest of the data for training, and compared performance of gene, population structure, and combined models to random selection of equally-sized test data (S7 Fig). For drugs with a known causative resistance gene, (i.e. every drug except CIP), adding gene information increases prediction accuracy by an average 0.24. The improvement is more pronounced if the resistance accessory gene has high penetrance and arises independently across different lineages (e.g. the trimethoprim resistance caused by the *dfrA* genes). No accessory gene is responsible for ciprofloxacin resistance and ST131 is highly resistant to ciprofloxacin. As a result, models with accessory gene and structure inputs perform equally well. Further, it is worth noting that the control accuracy (where held-out isolates were randomly sampled) was on average 0.28 higher for all drugs excluding AMP, AMX and CET, mainly due to higher sensitivity (S7 Fig). For these remaining antibiotics, the performance on the held-out clade was as good as for control. This is likely explained by more than 90% of the clade strains being resistant, and any clade correlate, either accessory gene presence, or population structure indicator, enabling accurate prediction. Overall, the results show that the accessory gene content may be used both for its genetic content of causative resistance determinants, and a proxy for structure, although the performance can worsen on novel lineages.

While the major mechanism for evolving antibiotic resistance is gene acquisition, mutations and indels on chromosomes may also play a role, and therefore aid prediction. We thus next included single nucleotide polymorphism (SNP) and indel data for gradient boosted decision trees and re-fitted the model. Predictive performance improved for 8 of the 11 antibiotics, and significantly so for cefuroxime and ciprofloxacin (S4B Fig). As anticipated, the largest accuracy improvement of 2.8% (C.I.: 1.61%-3.85%) occurred for ciprofloxacin, resistance against which is known to involve chromosomal mutations [33, 34]. Accordingly, the three most important identified features were variants in chromosomal quinolone-resistance-determining regions of the genes encoding DNA gyrase (*gyrA*), and topoisomerase IV *parC*. For other antibiotics, the addition of SNP data either did not greatly improve or worsened prediction performance (S4B Fig, Discussion). The addition of indel information did not significantly improve prediction, perhaps due to low frequency of such mechanisms.

A possible limitation for applying machine learning methods to detect antibiotic resistance is the unavoidably small number of samples (1,936 in this study) compared to the number of features (~18,270 in this study after collapsing the fully correlated features). To better understand how this imbalance impacts performance, we simulated data from different sample sizes using a range of penetrances for a single resistance determinant. As anticipated, the performance of gradient boosted decision trees dropped when the penetrance of the resistance determinant decreased (S8 Fig). However, there was no reduction in prediction and recall for resistance upon decreasing the population size, even when using only 130 strains. Overall, these findings suggest that the large number of features relative to sample size does not impact model performance for high frequency causal genes.

The current methods employ rule-based models to predict resistance from a small number of known determinants. We used *srst2* [32] to identify known resistance genes for cephalosporins, penicillins, aminoglycosides, trimethoprim and ciprofloxacin, and used this to compare the performance of our models with the prediction based on the presence of known genes in two databases. Except for amoxicillin, machine learning models attained a higher accuracy for prediction that rule-based approaches (S9 Fig). This is mainly due to reducing false negatives for resistance, where no known resistance gene was detected in the resistant strains.

Finally, we utilized the information from rule-based methods to better understand prediction errors for our model (Table 2). There were up to 47 false positive resistance calls across different antibiotics for strains that carried known resistance genes (beta-lactams, aminoglycoside modifying enzymes and *dfr* genes from CARD and Resfinder databases), but were annotated as susceptible. Manual inspection confirmed that all of these genes were fully covered by sequence data, and almost identical to the known resistance genes. In a similar vein, up to 17 false negative resistance calls did not contain a known causal determinant according to the same databases. Further, up to 65 resistant strains were correctly marked as resistant by our model, but contained no known resistance gene (Table 2). These discrepancies may be explained by unknown mechanisms for resistance, phenotypic resistance testing error, or genomic sequence coverage. As neither approach was perfect, predictive models in combination with rule-based methods may help identify cases that require further analysis or repeating the susceptibility tests, ultimately leading to improved diagnostics and novel mechanisms.

**Table 2:** Comparison of prediction results with a rule-based models with Resfinder and CARD database of antibiotic resistance genes.

Antibiotic	Acc. ML	Acc. CARD	Acc. ResFinder	TP w/o Res. gene (CARD)	FN w/o Res. gene (CARD)	FP w Res. gene (CARD)	TP w/o Res. gene (ResFinder)	FN w/o Res. gene (ResFinder)	FP w Res. gene(ResFinder)
**AMP**	**0.93**	**0.88**	**0.72**	**1/118**	**1/5**	**3/5**	**25/118**	**3/5**	**2/5**
**AMX**	**0.89**	**0.85**	**0.69**	**2/116**	**8/14**	**7/9**	**12/116**	**3/14**	**5/9**
**AMC**	**0.81**	**0.6**	**0.73**	**16/49**	**16/32**	**15/31**	**48/49**	**31/32**	**1/31**
**CTZ**	**0.95**	**0.8**	**0.65**	**0/49**	**2/14**	**1/4**	**10/49**	**5/14**	**4/4**
**CTX**	**0.97**	**0.8**	**0.64**	**0/67**	**0/5**	**1/2**	**9/67**	**3/5**	**1/2**
**CXM**	**0.91**	**0.8**	**0.67**	**0/67**	**8/25**	**4/7**	**15/67**	**13/25**	**5/7**
**CET**	**0.81**	**0.71**	**0.67**	**4/85**	**9/17**	**6/11**	**24/85**	**8/17**	**5/11**
**GEN**	**0.97**	**0.64**	**0.63**	**0/48**	**4/11**	**1/1**	**1/48**	**4/11**	**1/1**
**TBM**	**0.93**	**0.64**	**0.62**	**0/38**	**1/7**	**3/3**	**0/38**	**1/7**	**3/3**
**TMP**	**0.89**	**0.74**	**0.86**	**9/62**	**8/10**	**3/5**	**4/62**	**2/10**	**5/5**
**CIP**	**0.93**	**0.76**	**0.75**	**63/68**	**17/17**	**3/9**	**65/68**	**17/17**	**0/9**

True positives (TP), false positives (FP) and false negatives (FN) from Table 1.

Moreover, the discrepancy between rule-based and machine learning methods is most pronounced for CIP and AMC resistance. This is not surprising, as the resistance gene for CIP determine relatively small increases in the MICs of quinolones and are not considered in resistance screening [35]. Despite genome sequence information not being complete for any of the strains, our best performing model attained an accuracy of 0.93. This was achieved due to the use of genetic information correlated with resistance genes. For AMC resistance, *bla-TEM* gene was the most important feature used in prediction. However, not all variants of *bla-TEM* are known to exhibit inhibitor resistance [36]. In this case, prediction based on the presence of this gene may result in discrepant results with observed phenotypes. There results demonstrate that when genetic information on a key resistance gene is missing or incomplete in some resistant strains, the use of correlated genetic information leads to a reduction in false negatives for resistance, and improves accuracy.

## Discussion

We examined the ability of four different machine learning methods to predict antibiotic resistance from pan-genome data in *E*.*coli*, without making assumptions about the underlying genetic mechanisms. Our tests revealed that accessory genome data is needed for high accuracy in general, but that population structure information alone also allows prediction well above chance. This is particularly helpful when the genetic relatedness is known from a novel strain, whilst the underlying genetic resistance mechanism is unknown or less well-known, such as tend to be the case for recently introduced antibiotics. The quantification of the contribution of the population structure features remains out of the scope of this study.

Our input dataset was diverse. The collection comprised seven sequence types and strains from 15 consecutive years across a range of geographical locations. The majority of isolates (1509 of 1936 samples) were from a nationwide study across hospitals in the United Kingdom and Ireland [17], and associated with bacteremia. This geographical bias is not expected to affect the performance of the model on a new clinical dataset, since *E*. *coli* sequence types (e.g. ST131 clone [37]) are circulated across hospitals worldwide. However, as isolates from potential reservoirs, including hospital sewage and wastewater treatment plants, were underrepresented in training data (99 of 1936 samples), we cannot conclusively assess how well the trained models detect resistance in samples from these sources. More data are needed to develop robust models across the entire species range, especially if resistance mechanisms differ in the various niches.

The phenotype data was binary—each isolate was deemed either resistant or not to a compound. It is clear that this is an oversimplification of reality, as substantial variation hides within both categories. As evolutionary pressure is applied to the resistant population through the use of antibiotics, it will influence how quickly it spreads within and between patients, as well as in bacterial populations at large. To predict treatment outcomes, correctly design interventions and allocate societal resources, it will therefore be important to be able to accurately predict resistance quantitatively as well. This requires non-binary resistance data, acquired at high accuracy and throughput.

We compared five models, including a rule-based standard. As a baseline machine learning approach, we employed logistic regression, and contrasted it against arguably the most useful current methods. Indeed, deep neural networks, random forests, and gradient boosting form the top three most frequent winners in Kaggle (the world's largest community of data scientists and machine learners) competitions (https://www.kaggle.com/antgoldbloom/what-algorithms-are-most-successful-on-kaggle). In this regard, our findings confirm the utility of ensemble methods, and in particular boosting models, for predicting antibiotic resistance. While deep learning models are able to capture higher order interactions between features, and therefore often outperform simpler alternatives [38], they did not provide additional advantage here. Tree-based methods are often used as an intermediate between simple models that treat features independently, like logistic regression, and more complex, but poorly interpretable models. Indeed, random forest readouts can be analysed for feature importance as we have done here, and even detecting genetic interactions (e.g. [39]).

While the most frequently used features often captured information about known resistance genes, we do not attempt to interpret the identified links as causal. As for association methods [30, 40], the true impact of genetic features is confounded by their phylogenetic distribution and population structure. Therefore, approaches to distinguish causal resistance genes from all correlated markers require additional experimental study.

Recent reports have confirmed the strength of tree-based methods for predicting clinical attributes. For example, Wheeler *et al*. used random forests to predict invasiveness of *Salmonella enterica* lineages [16]. In another study, a tree ensemble was trained with boosting to predict the minimum inhibitory concentration from DNA k-mers for a large-scale *Klebsiella pneumoniae* panel [12], but the value of using core genome compared to accessory genes was not investigated. A very recent study [11] employed a different set of machine learning methods to predict resistance phenotype from pan-genome data but this study was conducted a small data set without testing gradient boosting and neural networks.

Including variant data or k-mers in the model greatly increases the number of features. However, adding the ~1 million additional single nucleotide features to ~20,000 others did not improve the results for most drugs in our dataset. This suggests that for *E*. *coli* collections or similar Enterobacteriaceae, in which resistance is driven to a great extent by horizontal transfer of entire genes, only pan-genome data may be directly useful in early screenings for resistance, and including nucleotide-level information may become more beneficial in the future, once causality is established for a broader range of genes, SNPs and indels. Furthermore, we expect methods that take population structure features as input to perform better in clonal organisms like *E*. *coli* compared with more recombinogenic species. The relative contributions of SNPs and population structure features to prediction will likely vary when methods are applied to different pathogens.

What is keeping predictive models from reaching 100% accuracy? One of the reasons for limited improvement due to SNP data could be their high false negative rate. Our model will fail to identify SNPs in regions that are too large for SNP detection, or SNPs that do not occur in the core genome, i.e. in the accessory genome. Furthermore, several non-genetic causes may influence predictive ability. The genome-based prediction cannot account for various non-genomic resistance mechanisms, such as resistance due to biofilm formation, or alteration of methylation patterns. Consequently, future studies should assess the value of even broader data for accurate prediction, ranging from transcriptome and proteome to other clinical and epidemiological data, such as cross-resistance and history of antibiotic therapy. Integrating these information sources from large isolate panels into a single predictive framework will lead to a rational basis for introducing machine learning in decision-making in public health.

## Supporting information

S1 Fig Phylogenetic distribution of resistance and Sequence Types (ST)s across the phylogenetic tree. A) A neighbor-joining phylogenetic tree from SNPs with associated resistance and susceptibility, major STs, and year of isolation information. B) Number of resistant (dark red), intermediate (light red) and susceptible (blue) strains (y-axis) for each of the 11 considered antibiotics (x-axis).
(TIF)

S2 Fig Comparison of the accuracy of prediction on the training dataset and the held-out data set. Bars with dark colors show the mean accuracy for the tuned model with 4-fold cross validation on the training dataset. The error bars are standard deviations. Bars with light colors are the accuracy of the tuned model on the held-out (test) dataset.
(TIF)

S3 Fig Prediction performance of the best tuned models. The precision (panels 3,4) and recall (panels 1, 2) for resistance and susceptibility for the best tuned predictive models on held out data for four predictive models (red: gradient boosted random forests; green: logistic regression; teal: random forests; purple: deep learning) across eleven antibiotics (x-axis). The best model, i.e. model with highest accuracy for resistance, of each class for every drug (x-axis) employed a number of possible combinations of gene presence, population structure, and year of isolation (lower panel; black: feature used; white: feature not used).
(TIF)

S4 Fig Improvement in the prediction performance. This was measured as increase or decrease in accuracy scores for resistance, after the inclusion of A) gene presence-absence (G) and population structure (S) input data and year of isolation (Y). B) SNP and indel data. Each bar shows the difference (extended feature set performance minus original feature set performance) between the mean accuracy scores for best performing models on the training data, with 4-fold cross validation. The error bars show the harmonic mean of standard deviation values for the two compared conditions, as defined in S3 Fig.
(TIF)

S5 Fig Feature importance analysis for the gradient boosted decision trees results. A) Average ranking (y-axis) and average importance (x-axis) of each feature across 50 random restarts of training for each feature (markers; size proportional to the frequency of feature utilization) for ceftazidime (CTZ). Annotations for top genes is shown in the figure. A full list of the genes is provided in S2 Table. B) Frequency (y-axis) of the year (green), population structure (blue) and gene presence (red) features for 100 most important features (x-axis) in the 11 antibiotics (panels) across the 50 random training restarts. The best performing model inputs, shown in Fig 1, are denoted to the right of each plot.
(TIF)

S6 Fig Prediction information of population structure data. Accuracy score for prediction of antibiotic resistance from population structure alone (y-axis) for randomized labels (violin plots) and real data (red marker) across 11 antibiotics (x-axis). The violin plots aggregate results from 100 bootstrap replicates of randomly sampled phenotype labels. A gradient boosted decision trees model with 600 iterations was used as predictive model.
(TIF)

S7 Fig Assessment of the performance of model with different features on a new lineage for 11 drugs. We left out ST131 strains as test data set and trained the model on the rest of strains (w/o ST131). Results are compared with a control case, in which test and train datasets with the same size as w/o ST131 were created by a random selection (Control). Models were tuned with three features combinations, i.e. S (population structure), G (Accessory genes) and G+S (Accessory genes and population structure). The first row shows frequency of resistant strains in the train and test datasets for the two cases. The second panel shows the accuracy for predicting resistance and the difference between the accuracy values, i.e. Accuracy_control_-Accuracy_w/o ST131_. The two last panels show precision and recall for resistance with three feature combinations.
(TIF)

S8 Fig Effect of sample size on the performance of the predictive model. Precision (left panel) and recall (right panel) for resistance phenotype for different population sizes (colors) and penetrances (x-axis) in simulated pan-genome data using gradient boosted decision trees.
(TIF)

S9 Fig Comparison between rule based and machine learning methods. Results from Rule based methods with two databases of known resistance genes, i.e. ResFinder and CARD, were compared with results from the best performing models from our study for 11 drugs. We evaluated the performance on the held-out dataset used in assessing predictive models.
(TIF)

S1 Table List of isolates with associated metadata and accession numbers in the European Nucleotide Archive (ENA). (CSV)

S2 Table List of important accessory genes and their functions for feature importance analysis with the best performing gradient boosted decision trees models shown in S4 Fig. The importance metrics include the number of runs (total 50 runs), in which the feature was used during model optimization and the average ranking and importance for the feature in these runs. Sequences for the genes are provided in the Github directory (see Methods).
(CSV)

S3 Table Prediction results for the best performing model for 11 drugs. (CSV)
