# Supplementary figures and images for: Prediction of antibiotic resistance in Escherichia coli from large-scale pan-genome data
Source: PLoS Comput Biol. 2018 Dec 14;14(12):e1006258. doi: 10.1371/journal.pcbi.1006258 (PMC6310291; doi:10.1371/journal.pcbi.1006258)

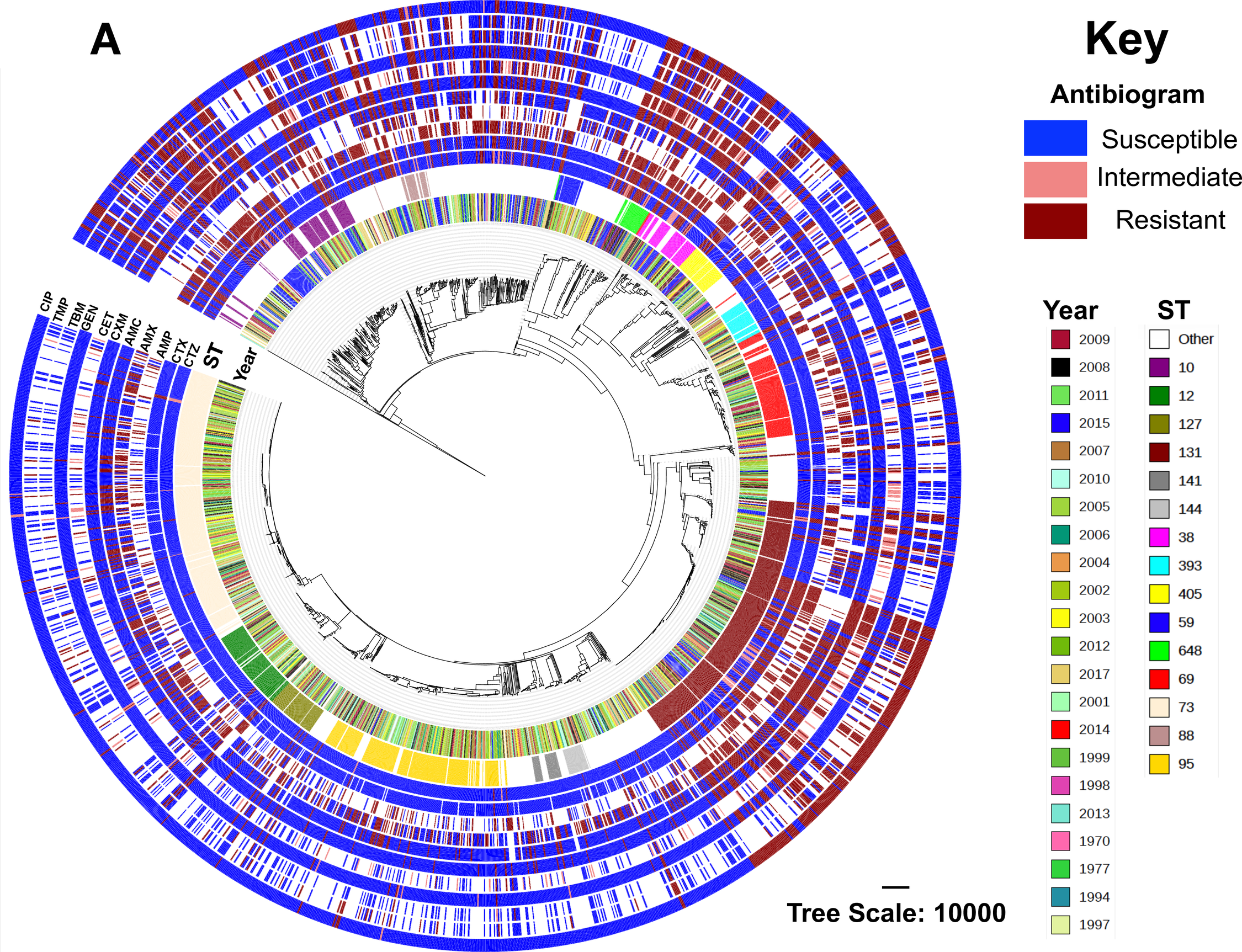

Supplement: S1 Fig — A) A neighbor-joining phylogenetic tree from SNPs with associated resistance and susceptibility, major STs, and year of isolation information. B) Number of resistant (dark red), intermediate (light red) and susceptible (blue) strains (y-axis) for each of the 11 considered antibiotics (x-axis). (TIF) [file pcbi.1006258.s001.tif]

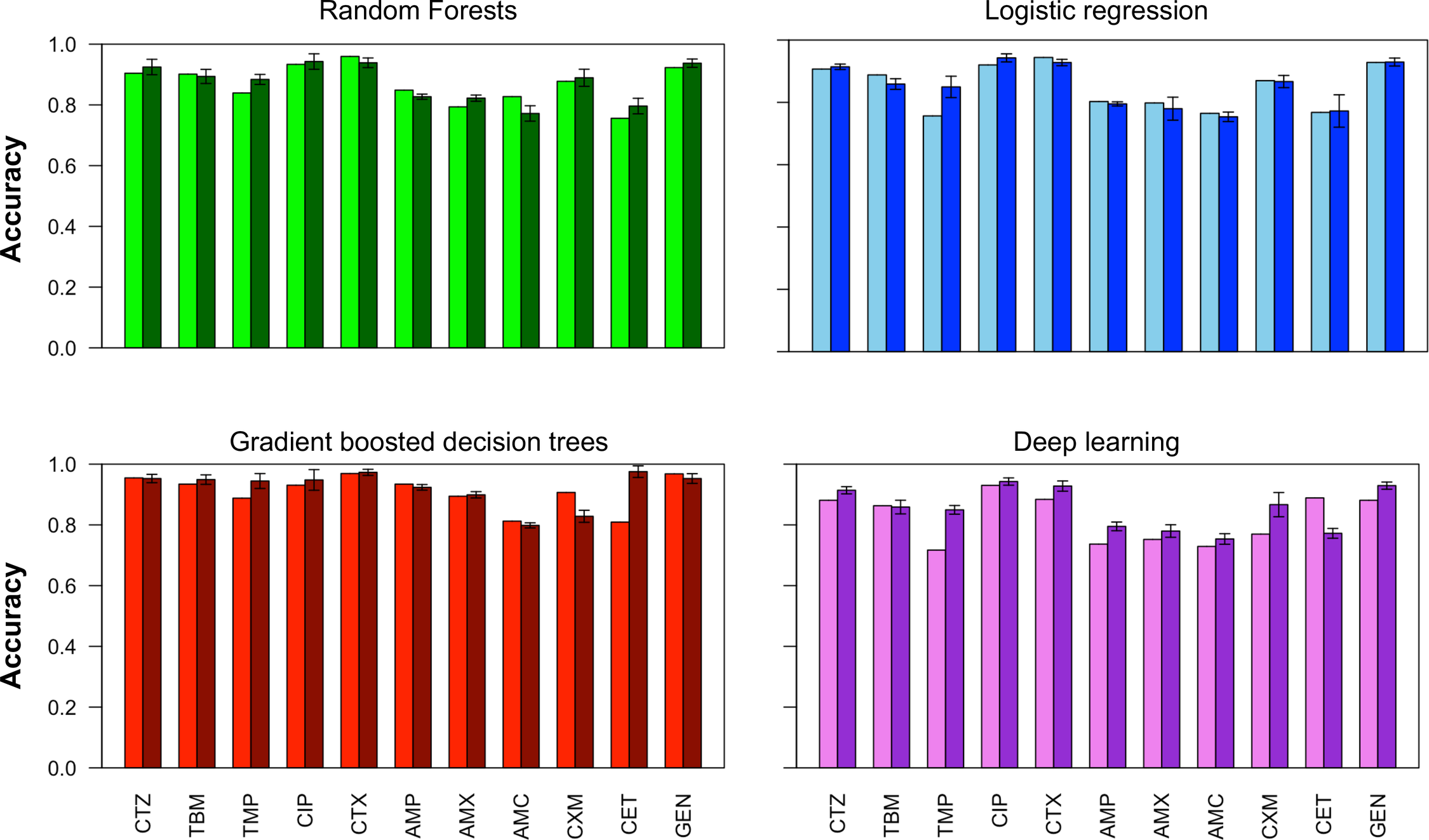

Supplement: S2 Fig — Bars with dark colors show the mean accuracy for the tuned model with 4-fold cross validation on the training dataset. The error bars are standard deviations. Bars with light colors are the accuracy of the tuned model on the held-out (test) dataset. (TIF) [file pcbi.1006258.s002.tif]

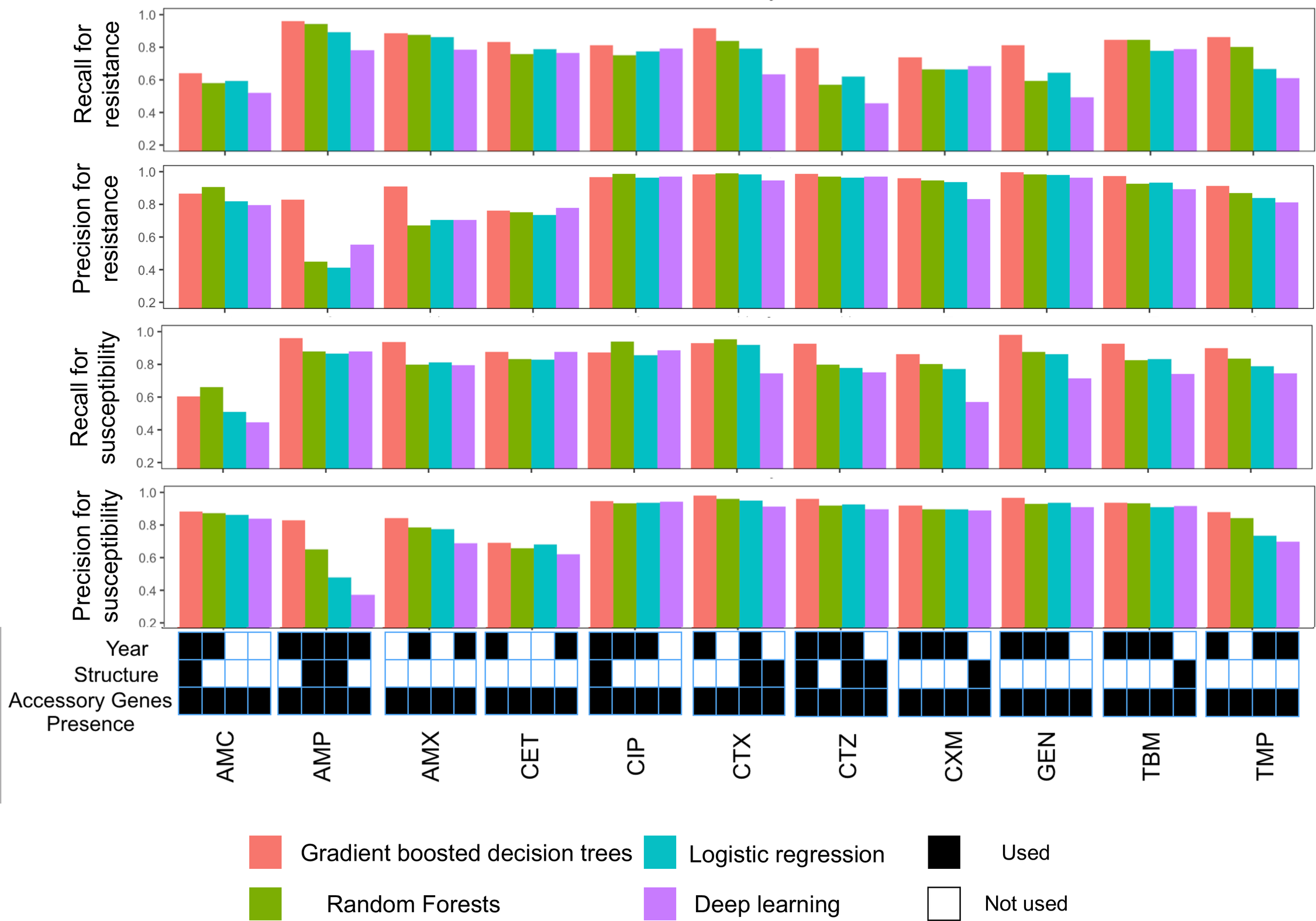

Supplement: S3 Fig — The precision (panels 3,4) and recall (panels 1, 2) for resistance and susceptibility for the best tuned predictive models on held out data for four predictive models (red: gradient boosted random forests; green: logistic regression; teal: random forests; purple: deep learning) across eleven antibiotics (x-axis). The best model, i.e. model with highest accuracy for resistance, of each class for every drug (x-axis) employed a number of possible combinations of gene presence, population structure, and year of isolation (lower panel; black: feature used; white: feature not used). (TIF) [file pcbi.1006258.s003.tif]

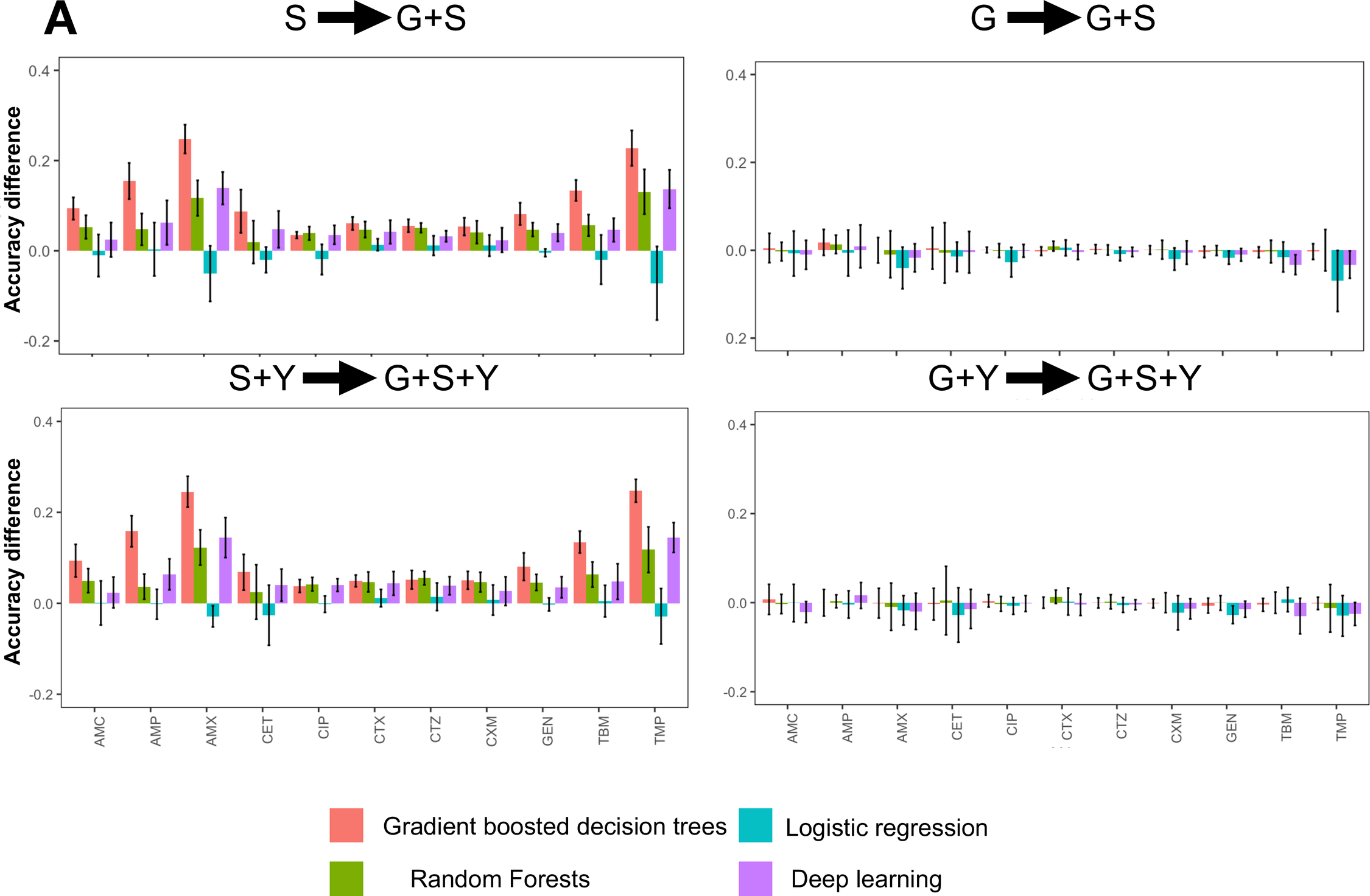

Supplement: S4 Fig — This was measured as increase or decrease in accuracy scores for resistance, after the inclusion of A) gene presence-absence (G) and population structure (S) input data and year of isolation (Y). B) SNP and indel data. Each bar shows the difference (extended feature set performance minus original feature set performance) between the mean accuracy scores for best performing models on the training data, with 4-fold cross validation. The error bars show the harmonic mean of standard deviation values for the two compared conditions, as defined in S3 Fig. (TIF) [file pcbi.1006258.s004.tif]

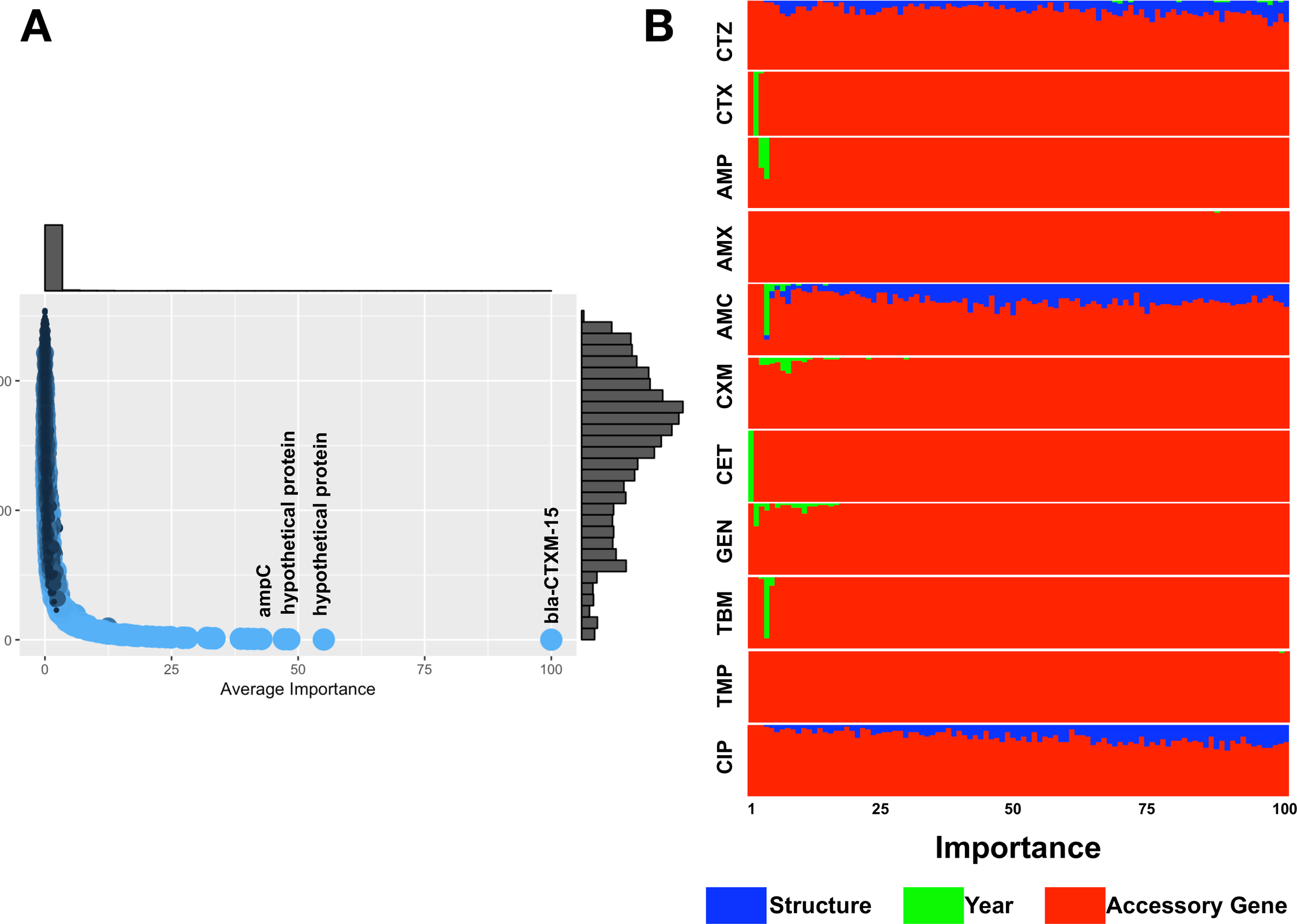

Supplement: S5 Fig — A) Average ranking (y-axis) and average importance (x-axis) of each feature across 50 random restarts of training for each feature (markers; size proportional to the frequency of feature utilization) for ceftazidime (CTZ). Annotations for top genes is shown in the figure. A full list of the genes is provided in S2 Table. B) Frequency (y-axis) of the year (green), population structure (blue) and gene presence (red) features for 100 most important features (x-axis) in the 11 antibiotics (panels) across the 50 random training restarts. The best performing model inputs, shown in Fig 1, are denoted to the right of each plot. (TIF) [file pcbi.1006258.s005.tif]

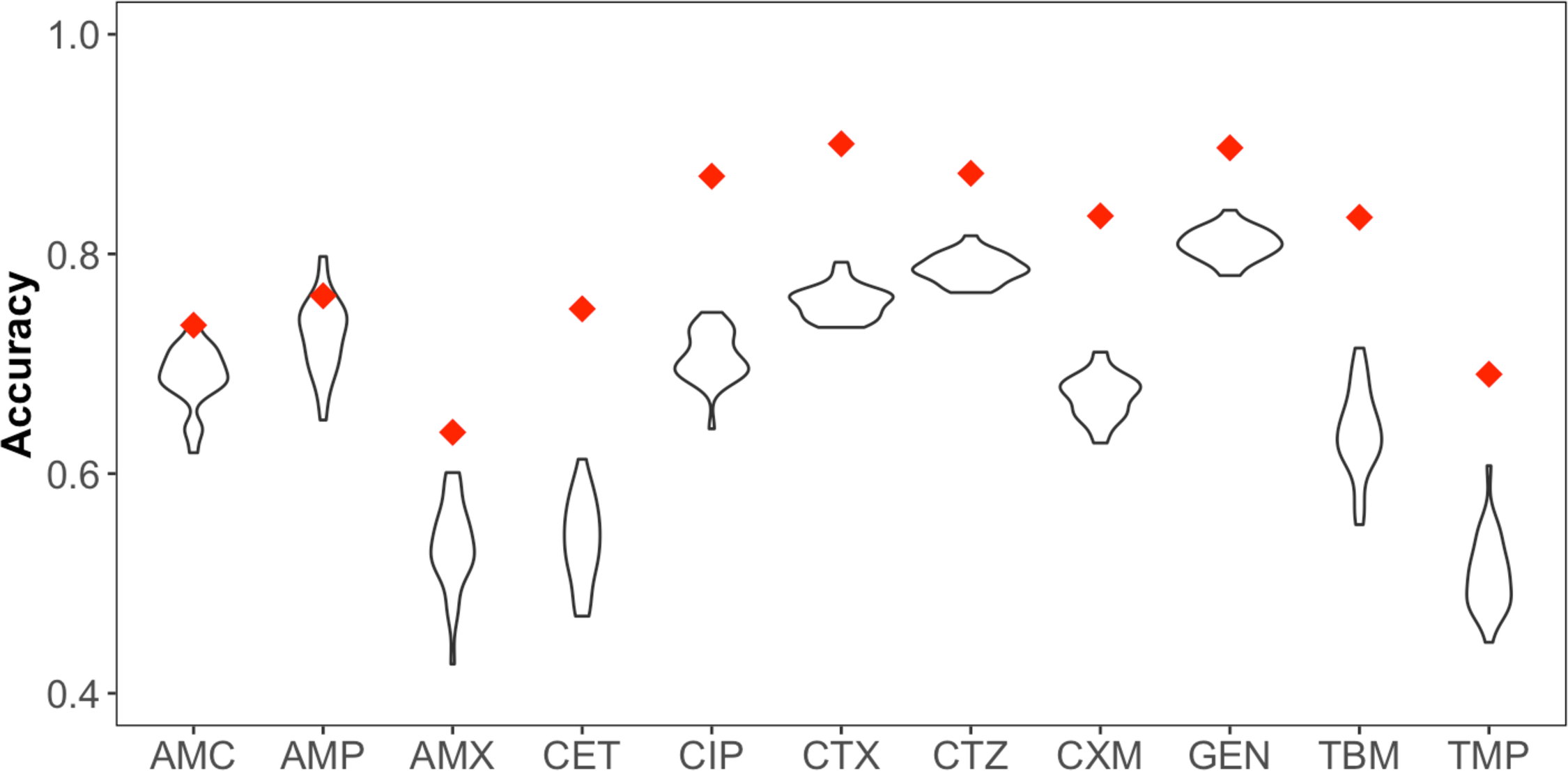

Supplement: S6 Fig — Accuracy score for prediction of antibiotic resistance from population structure alone (y-axis) for randomized labels (violin plots) and real data (red marker) across 11 antibiotics (x-axis). The violin plots aggregate results from 100 bootstrap replicates of randomly sampled phenotype labels. A gradient boosted decision trees model with 600 iterations was used as predictive model. (TIF) [file pcbi.1006258.s006.tif]

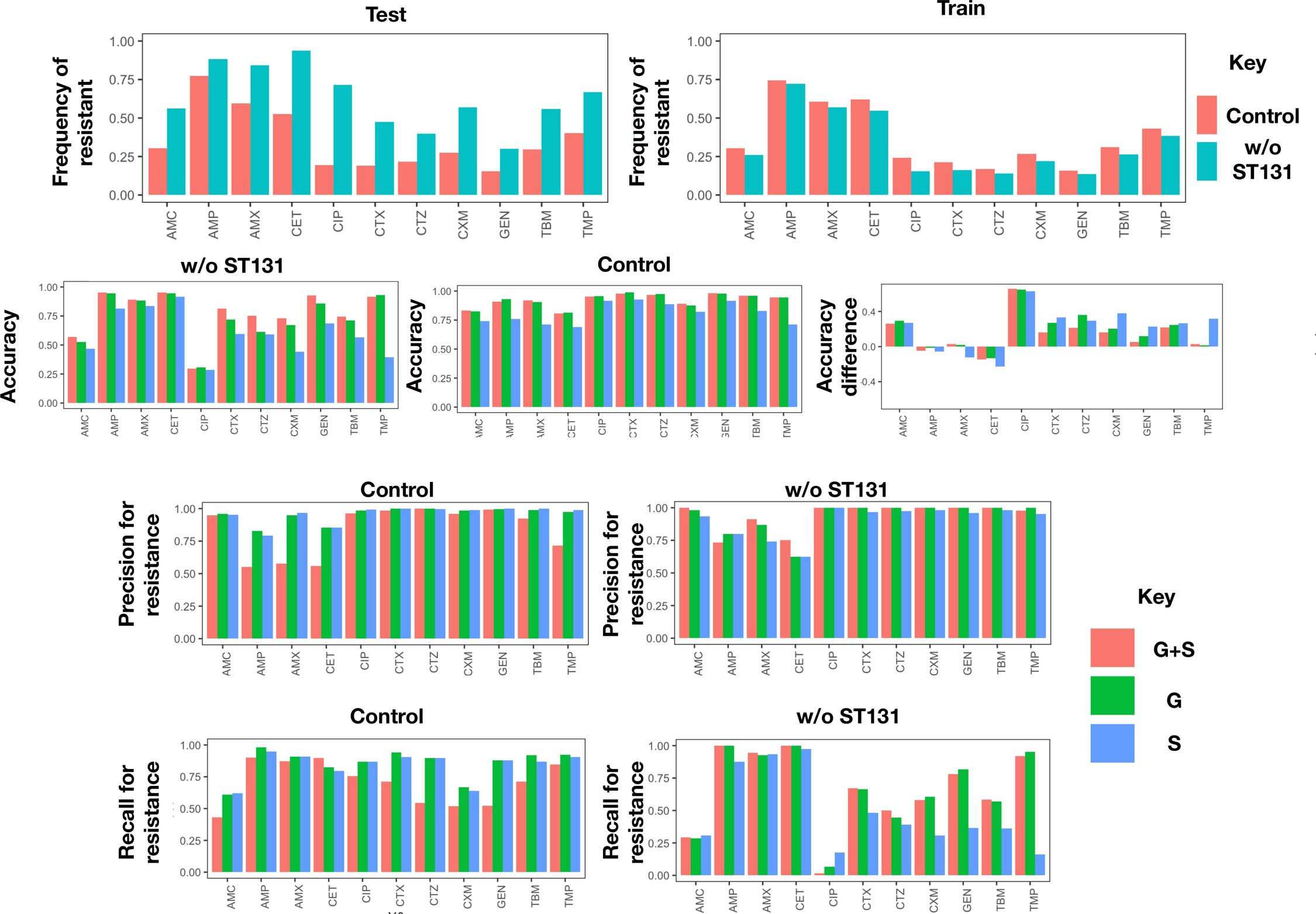

Supplement: S7 Fig — We left out ST131 strains as test data set and trained the model on the rest of strains (w/o ST131). Results are compared with a control case, in which test and train datasets with the same size as w/o ST131 were created by a random selection (Control). Models were tuned with three features combinations, i.e. S (population structure), G (Accessory genes) and G+S (Accessory genes and population structure). The first row shows frequency of resistant strains in the train and test datasets for the two cases. The second panel shows the accuracy for predicting resistance and the difference between the accuracy values, i.e. Accuracycontrol-Accuracyw/o ST131. The two last panels show precision and recall for resistance with three feature combinations. (TIF) [file pcbi.1006258.s007.tif]

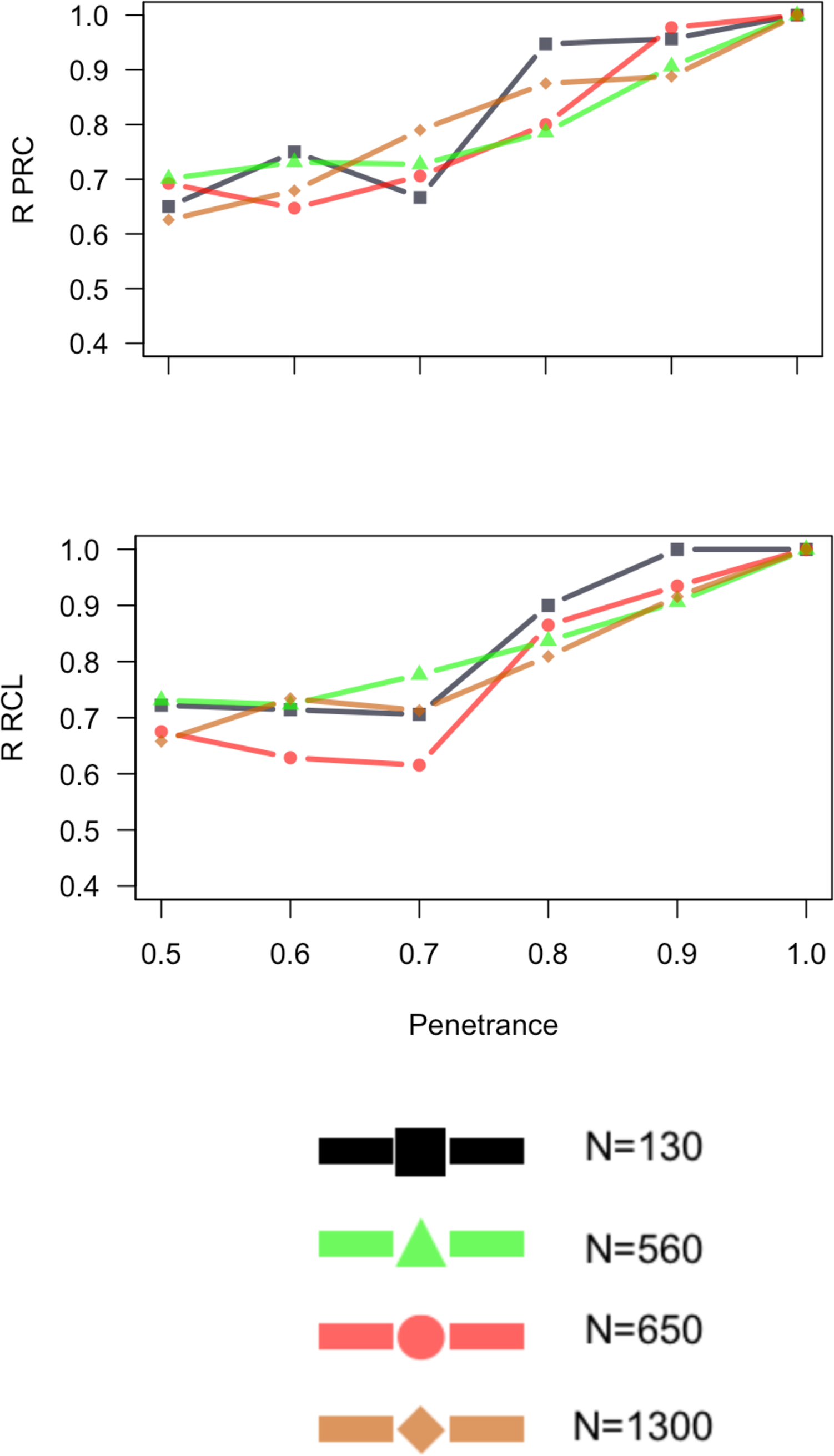

Supplement: S8 Fig — Precision (left panel) and recall (right panel) for resistance phenotype for different population sizes (colors) and penetrances (x-axis) in simulated pan-genome data using gradient boosted decision trees. (TIF) [file pcbi.1006258.s008.tif]

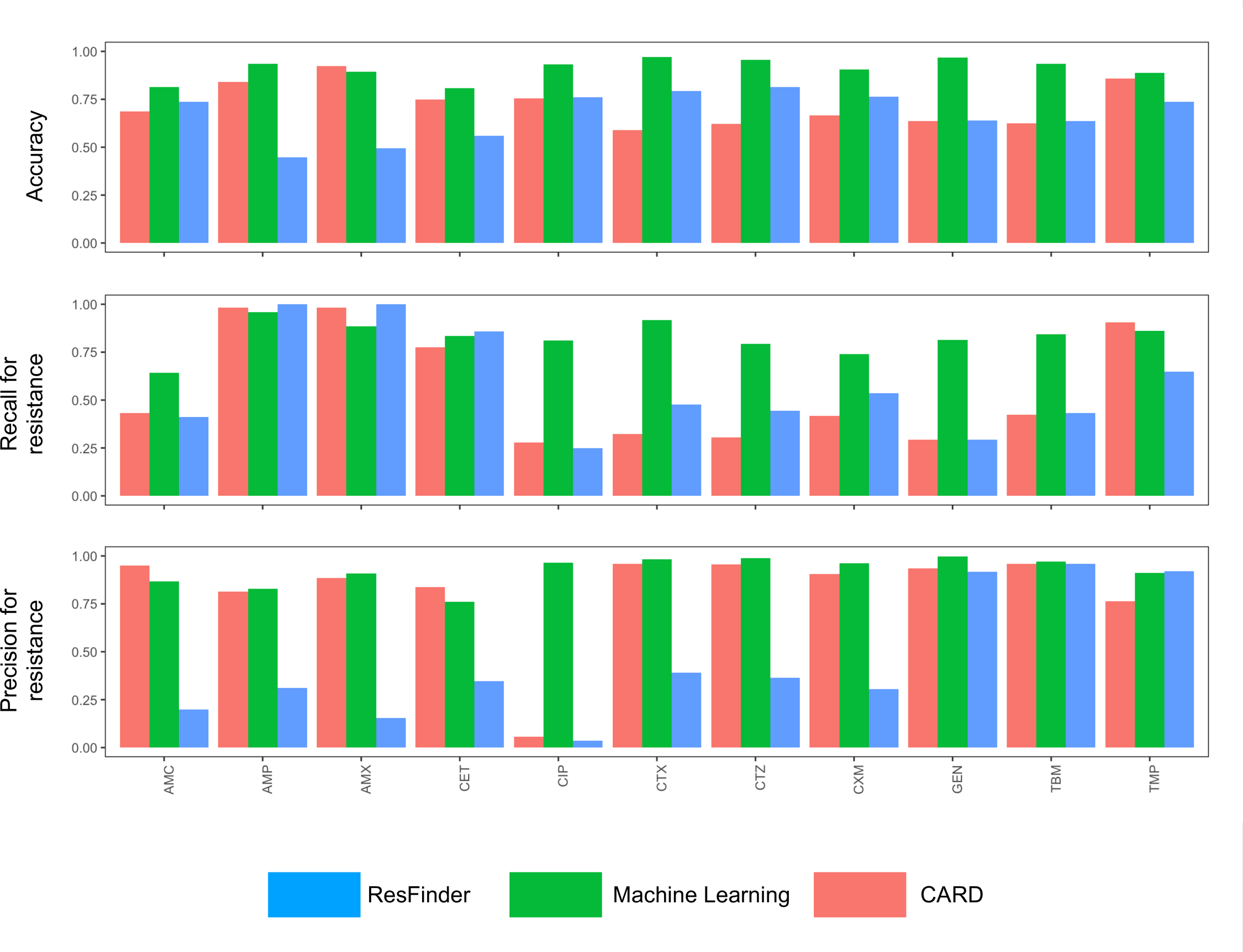

Supplement: S9 Fig — Results from Rule based methods with two databases of known resistance genes, i.e. ResFinder and CARD, were compared with results from the best performing models from our study for 11 drugs. We evaluated the performance on the held-out dataset used in assessing predictive models. (TIF) [file pcbi.1006258.s009.tif]
